# Autologous matrix induced chondrogenesis (AMIC) as revision procedure for failed AMIC in recurrent symptomatic osteochondral defects of the talus

**DOI:** 10.1038/s41598-022-20641-6

**Published:** 2022-09-28

**Authors:** Filippo Migliorini, Hanno Schenker, Nicola Maffulli, Jörg Eschweiler, Philipp Lichte, Frank Hildebrand, Christian David Weber

**Affiliations:** 1 Department of Orthopaedic, Trauma, and Reconstructive Surgery, RWTH University Hospital, Pauwelsstraße 31, 52074 Aachen, Germany; 2 Department of Medicine, Surgery and Dentistry, University of Salerno, 84081 Baronissi, SA Italy; 3 School of Pharmacy and Bioengineering, Faculty of Medicine, Keele University, ST4 7QB Stoke On Trent, England; 4 Barts and the London School of Medicine and Dentistry, Centre for Sports and Exercise Medicine, Mile End Hospital, Queen Mary University of London, E1 4DG London, England

**Keywords:** Medical research, Translational research

## Abstract

Autologous matrix induced chondrogenesis (AMIC) is a bone marrow stimulating technique used for the surgical management of chondral defects of the talus. The present study evaluated the clinical outcomes and imaging of AMIC as revision procedure for failed AMIC surgery for osteochondral defects of the talus. Forty-eight patients with symptomatic osteochondral defects who received a revision AMIC were evaluated after a minimum of two years follow-up. Patients with previous procedures rather than AMIC, those who required additional surgical procedures (e.g. ligament repair or deformity correction), or those who had evidence of kissing, bilateral, or multiple lesions were excluded. Outcome parameters included the Visual Analogic Scale (VAS), Tegner Activity Scale, the American Orthopedic Foot and Ankle Score (AOFAS), and the Magnetic Resonance Observation of Cartilage Repair Tissue (MOCART) score. All patients were followed by an assessor who was not involved in the clinical management. 27 patients were enrolled in the present study. The mean age of the patient was 34.9 ± 3.1 years, and the mean BMI 27.2 ± 5.1 kg/m^2^. The mean defect surface area was 2.8 ± 1.9 cm^2^. The mean follow-up was 44.3 ± 21.4 months. The mean hospital length of stay was 4.4 ± 1.4 days. At final follow-up, the mean VAS score was 4.1 ± 3.1, the mean Tegner 3.5 ± 1.6, the mean AOFAS 58.8 ± 20.6. The preoperative MOCART score was 22.1 ± 13.7 points, the postoperative MOCART score was 42.3 ± 27.9 points (+ 20.2%; P = 0.04), respectively. 30% (8 of 27 patients) experienced persistent pain and underwent a further chondral procedure. Concluding, AMIC could be a viable option as revision procedure for failed AMIC in recurrent symptomatic osteochondral defects of the talus. The PROMs indicated that patients were moderately satisfied with the procedure, and the MOCART score demonstrated a significant improvement from baseline to the last follow-up. A deeper understanding in prognostic factors and patient selection is critical to prevent failures.

## Introduction

Focal chondral and osteochondral defects of the talus are common in sports medicine^1–3^. The etiology and long-term natural course of these lesions has not been fully elucidated; some studies suggest only moderate or even no progression of joint degeneration following non-operative management^4,5^, while others report a concerning progression to frank ankle osteoarthritis even in young patients^6^. Frequently, symptomatic osteochondral defects of the talus limit sports and daily activities, and may require surgical management^7^. Lesions refractory to non-surgical care for three to six months may be suitable for surgical management^8^.

For primary osteochondral talar lesions, a variety of surgical treatments has been proposed^9–15^, but the superiority of any surgical technique has not been confirmed^16^. For lesions smaller than 1.5 cm^2^, bone marrow stimulation (BMS) techniques provide satisfactory long-term outcomes^17^, and a pooled success rate of 61% was reported for non-primary lesions^18^. For larger lesions, both matrix-induced autologous chondrocyte implantation (mACI) and autologous matrix-induced chondrogenesis (AMIC) have been proposed^19^. Theoretically, mACI should induce a superior repair with type II cartilage, and could be ideal for both primary and revision settings. However, although mACI provides reliable outcomes, these seem to be equivalent to other surgical techniques, but at higher costs^20^. Osteochondral transplantation techniques have also been suggested as a revision modality for osteochondral lesions of the talus^21,22^. A major drawback of the technique is the associated donor site morbidity, especially if multiple plugs are harvested^23^. In recent studies, autograft plugs outperformed allograft tissues in terms of efficacy, chondral wear and the number of secondary procedures^24^. However, quantitative T2 mapping suggests that even autograft tissue may not mirror native hyaline cartilage^25^, indicating a potential cellular dedifferentiation of transplanted cartilage over time. Further problems are associated with graft integration and less than ideal restoration of anatomic joint congruence^25^. For these reasons, AMIC combined with bone grafting has gained further interest, as it might also present a feasible single-stage procedure for revisions, without sacrificing osteochondral tissue from the knee joint. Especially for larger non-primary lesions, published data remain sparse.

Whether repeat AMIC performed in revision settings is feasible has not been studied to date. The present study evaluated the clinical outcomes and imaging of AMIC as revision procedure for failed AMIC surgery for osteochondral defects of the talus. We hypothesised that AMIC could be a viable option as a revision procedure for failed AMIC surgery for osteochondral defects of the talus.

## Methods

### Patient recruitment

The present study was conducted according to the principles of the Declaration of Helsinki and was approved by the ethics committee of the RWTH Aachen University (project ID EK 305/13). The present study follows the Strengthening the Reporting of Observational Studies in Epidemiology: the STROBE Statement^26^. In May 2021 the databases of the RWTH University Clinic of Aachen (Germany) and University Hospital of Salerno (Italy) were accessed. All the patients who had undergone AMIC following failed previous AMIC for chondral defects of the talus were retrieved. The inclusion criteria were: (1) failed previous AMIC procedure for focal osteochondral defect, (2) pain lasting > 6 months, (3) failed conservative therapies, (4) pain suggestive of focal chondral defect, (5) have undergone preoperative MRI with suggestive findings, (6) minimum two years follow-up, and (7) signed informed consent. The exclusion criteria were: (1) other previous or concomitant procedures rather than isolated AMIC, (2) kissing lesions, (3) bilateral lesions, (4) multiple lesions, (5) any other relevant pathology that can influence the study, and (6) patients unable to understand the nature of the treatment of the study (e.g. cognitive impairment, language barrier).

### Surgical technique

All the surgeries were performed by three experienced surgeons following the same surgical protocol. The ankle was plantar flexed, and a 2 mm Kirscher-wire was drilled in the distal tibia and another one in the talus. A Hintermann spreader (Integra LifeSciences, Plainsboro, NJ) was used for joint distraction. A diagnostic arthroscopy was performed using standard anterolateral and anteromedial portals. The osteochondral defect was identified, and the necrotic surrounding cartilage was shaved until a viable chondral shoulder was reached. Subsequently, a malleolar osteotomy was performed. The subchondral bone was milled until active bleeding was achieved and the defect filled using autologous cancellous bone grafting harvested from the ipsilateral osteotomy site. Subsequently, the residual chondral defect was measured with a metal template. A collagen I/III porcine resorbable membrane (Chondroguide®, Geistlich Pharma AG, Switzerland) was accurately trimmed to slightly oversize the defect. Fibrin glue was used to secure the membrane into the defect. Two malleolar screws were used to fix the malleolar osteotomy. The surgical wound was sutured in a standard fashion. The ankle was flexed and extended several times and the correct positioning of the membrane was confirmed arthroscopically. The postoperative care and rehabilitation protocols followed were conducted according to previously published reports^27,28^.

### Outcomes of interest

On admission, the following data were recorded: age, gender, side, date of surgeries, additional autologous cancellous bone grafting during the index procedure, BMI (Kg/m^2^), symptoms duration prior of the revision surgery and length of the hospitalisation. The area of the defect was measured using MRI sequences^29^. In May 2021, with informed consent, patients were invited to complete the following patient reported outcome measures (PROMs): Visual Analogic Scale (VAS), Tegner Activity Scale, and the American Orthopaedic Foot and Ankle Score (AOFAS). Data on complications and additional procedures were also retrieved. A MRI was performed to every patient at follow-up. The MRI results were evaluated using the Magnetic Resonance Observation of Cartilage Repair Tissue (MOCART) score^30^. The MOCART score was assessed by a trained radiologist with experience in musculoskeletal imaging and successively double checked by an experienced orthopaedic surgeon. Disagreements between the two assessors were debated and solved by discussion. Patients who did not agree to participate in the clinical assessment and/or imaging examination were excluded from the present investigation. All the patients were followed by an assessor who was not involved in the clinical management of the patients and blinded to MRI results.

### Statistical analysis

All statistical analyses were performed by the main author (F.M.) using the software IBM SPSS (Version 25). Continuous variables were analysed with mean difference (MD) and standard error (SE). The confidence interval (CI) was set at 95%. Values of t-test < 0.05 were considered statistically significant.

### Ethical approval

The present study was conducted according to the principles of Helsinki and was approved by the ethic committee of the RWTH Aachen University (project ID EK 305/13).

### Consent to participate

All patients firmed written consent and willingness to participate to the present study.

## Results

### Patient recruitment

**Figure 1:**
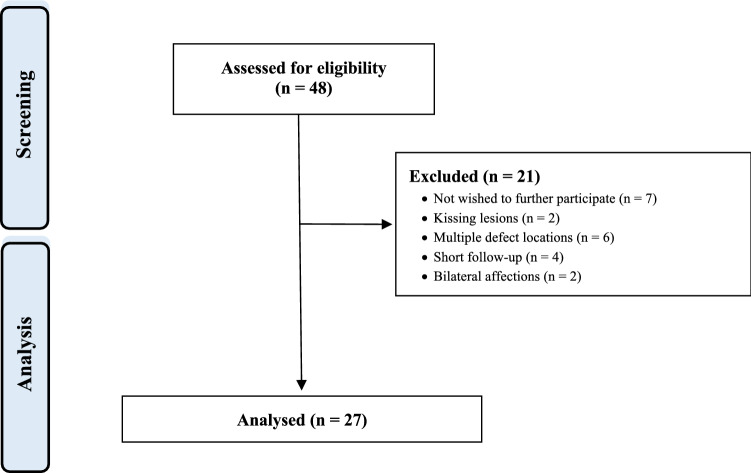
Diagram of the recruitment process.

A total of 48 patients underwent repeat AMIC following failure of previous AMIC of the talus. A total of 14 patients were excluded as they did not match the eligibility criteria: kissing lesions (N = 2), bilateral lesions (N = 2), multiple lesions (N = 6), chronic disease which may influence the outcome (N = 1), follow-up shorter than two years (N = 4). A further 7 patients were excluded because they did not wish to participate in the study. Eventually, 27 patients were enrolled in the present study (Fig. 1).

### Patient demographic

63% (17 of 27 patients) were female. The mean time span between the index and the revision surgery was 18.3 ± 5.7 months. The mean follow-up was 44.3 ± 21.4 months. The mean area of the defect was 2.8 ± 1.9 cm^2^. 63% (17 of 27) of defects were medial, and 10 of 27 were lateral. During the index procedure, 44% (12/27) of patients received an additional autologous cancellous bone graft from the osteotomy site. Patient demographic data are shown in greater detail in Table 1.

**Table 1:** Demographic data of the patients before the revision surgery.

Endpoint	Value
Follow-up (months)	44.3 ± 21.4
Age	34.9 ± 3.1
Women	63% (17 of 27)
Length of the symptoms (months)	41.6 ± 59.6
Length of the hospitalisation (days)	4.4 ± 1.4
MOCART score	22.1 ± 13.7
Area of defect (cm^2^)	3.2 ± 1.9
BMI (kg/m^2^)	27.2 ± 5.1

### Outcomes of interest

At last follow-up, the mean VAS score was 4.1 ± 3.1, the mean Tegner 3.5 ± 1.6, the mean AOFAS 58.8 ± 20.6 (Table 2).

**Table 2:** Main results at last follow-up.

Endpoint	Value	Range
AOFAS	58.8 ± 20.6	28–77
VAS (0–10)	4.1 ± 3.1	0–8
Tegner activity scale	3.5 ± 1.6	2–6

The MOCART score reached 42.3 ± 27.9 points (MD + 20.2%; SE 5.379; 95% CI 9.41–30.98; P = 0.04, Table 3).

**Table 3:** Results of the MOCART score for each item (FU: follow-up).

Items	Mean values	
	At baseline	At FU
Degree of defect repair and defect filling (0–20)	4.9 ± 4.1	7.9 ± 5.6
Cartilage interface (0–15)	3.3 ± 2.5	6.7 ± 4.9
Surface of the repaired tissue (0–10)	1.8 ± 1.9	4.2 ± 2.1
Structure of the repaired tissue (0–5)	1.1 ± 0.8	2.7 ± 2.3
Signal intensity (0–30)	5.0 ± 6.7	10.1 ± 9.0
Subchondral lamina (0–5)	1.9 ± 2.9	2.4 ± 1.9
Subchondral bone (0–5)	1.7 ± 1.9	2.7 ± 1.1
Adhesions (0–5)	1.3 ± 1.2	3.1 ± 1.2
Effusion (0–5)	1.1 ± 1.9	2.5 ± 2.4

One patient experienced a wound infection, which was successfully treated with antibiotics. 30% (8 of 27 patients) experienced persistent pain, and underwent a further chondral procedure. All the patients underwent screws removal at a minimum of 12 months postoperatively. No complications related to the osteotomy were reported.

## Discussion

According to the main findings of the present study, repeat AMIC combined with cancellous bone graft may be a viable treatment option for larger (mean 3.2 cm^2^) osteochondral lesions of the talus in patients in whom a primary AMIC procedure failed. Current evidence on revision procedures following failed surgical management for osteochondral defects is limited, and unpredictable results are reported. The clinical outcomes and imaging of AMIC as revision procedure for a failed AMIC for osteochondral defects of the talus have been scantly investigated. To the best of our knowledge, this is the first patient series detailing the results of repeat AMIC for chondral and osteochondral lesions of the talus.

The single most important indication for reoperation following a repeat AMIC was implant removals after medial malleolar osteotomy. This finding agrees with a recent systematic review, reporting that tissue irritation requiring hardware removal was the most frequent adverse event of the index procedure^31^. In the current literature, the re-intervention rate of the open AMIC was reported to be 9 to 14%, and age, BMI, and the preoperative performance status of the patients seem to have a detrimental impact on clinical outcomes after surgical treatment of osteochondral lesions of the talus^11,28,31–34^. The impact of biological (e.g. age), lesion-specific (location, size, acuity) and biomechanical (e.g. BMI, instability) variables have been reported as prognostic factors^35^. Yoon et al. suggested that 11% of 399 patients after primary arthroscopic treatment required revision surgery. These data suggest that there is a significant number of secondary and tertiary procedures after failed surgical treatment of osteochondral lesions around the ankle. Unfortunately, the level of evidence of the published reports remains low. AMIC combined with autologous bone grafting can often be performed through an anterolateral or anteromedial arthrotomy, avoiding a malleolar osteotomy^36^. Furthermore, an all arthroscopic AMIC has been described for talar osteochondral defects^37^. These recent advances may help to further reduce the number of re-interventions. Lambers et al. evaluated 21 studies (n = 299 patients) with 301 talar OCDs that failed primary surgery^38^. The treatment strategies included conservative treatment, BMS, retrograde drilling, osteochondral transplantation, cartilage implantation and chondrogenesis-inducing therapies (CIT). Two studies based on AMIC were included, and the calculated success rates were 67% (CI 30–90.3%) and 57% (CI 31.6–78.6%), respectively. In our cohort, 70% of patients required no further procedure addressing the osteochondral defect. In a study evaluating AMIC as both primary and revision procedures, Kubosch et al. reported arthroscopic debridement, retrograde drilling and ligament reconstruction as primary procedures. Valderrabano et al. often combined AMIC with a corrective calcaneal osteotomy, when hindfoot malalignment (≥ 10°) was identified; furthermore, a modified Brostrom-Gould procedure was added in unstable ankles (17/26). Thus, concomitant pathologies and accessory procedures pose a significant impact on patient reported outcomes, and must be considered as confounding variables. We therefore included only patients who received a revision AMIC and had no concomitant procedures other than autologous cancellous bone graft harvested from the proximal tibia or iliac crest. In a recent study by the same group, AMIC demonstrated greater outcomes measures by AOFAS, VAS and Tegner scores at follow-up compared to isolated microfractures^9^. AMIC has been enhanced using bone marrow aspirate as an adjunct to type I/III type collagen matrix^39^. Murphy et al. evaluated the matrix-associated stem cell transplantation (MAST) technique applied in patients with larger lesions of the talar dome (15mm^2^ or greater) and patients with a previous failed attempt at microfracture (n = 21), AMIC (n = 1) or OATS (n = 1). The proposed advantage of this treatment method includes the delivery of mesenchymal stem cells right into the defect and a low donor site morbidity, when compared to ACI or OATS. However, the study did not include revision cases only, patients presented with relatively small defects (mean 1.7cm^2^), and lacks an MRI follow-up and a control group.

Up to date, for large lesions after failed treatment, no optimal surgical management has been established. For tertiary osteochondral defects, the use of contoured metal implants as salvage procedure has been suggested^40^. Maiorano et al. reported a series of 12 patients (mean age 39) after metal resurfacing (HemiCAP®; Arthrosurface Inc., Franklin, MA), and observed that pain was still present at follow-up^41^. They therefore concluded that metal resurfacing might not be considered as valid alternative for treatment of osteochondral defects after failed previous surgery. Ettinger et al. retrospectively evaluated a cohort of 10 patients (mean age 47 years, mean BMI 30), who received a HemiCAP® implantation of the medial talar dome after failed previous surgery^42^. The authors reported an increased risk for postoperative dissatisfaction and persistent ankle pain in overweight patients. Moreover, ten revisions were performed in 7 patients (70%)^42^. In contrast, a Danish group reported about 31 consecutive patients (mean age 42.8 years) after HemiCAP® implantation, and described one infection, but good mid-term results and no revisions, these latter defined as conversion to ankle fusion, total ankle prosthesis or revision of the implant^43^. Especially in young and active individuals, various issues including persisting pain and long-term concerns (implant survival) remain associated with (partial) joint replacement, suggesting that a biological treatment concept should be exploited first. For large primary and secondary osteochondral defects of the medial talus, Kerkhoffs et al. recently suggested the Talar OsteoPeriostic grafting from the Iliac Crest (TOPIC) procedure^10^. This new biological concept has the advantage to provide of a natural scaffold and to mimic the talus anatomically. The anatomic and press-fit incorporation of the graft may help to treat especially cystic lesions and to avoid peak pressures on the tibial plafond, when compared to metallic implants.

We acknowledge a number of strengths and limitations within our study. First, the cohort included only patients with a previously failed surgical treatment by an open AMIC. Previous studies often included cohorts with heterogeneous surgical first-line treatments or variable additional procedures. As a consequence of these strict inclusion criteria, the number of patients in our study is limited. The rate of patients who were lost at follow-up (7 of 34) may increase the risk of attrition bias and impact negatively our conclusion. However, the follow-up was conducted during the COVID-19 pandemic, and some patients did not wish to participate to the study. The AOFAS score has not been validated for patients with osteochondral defects around the talus. Also, a formal control group is not included and a second look arthroscopy has not been performed. Data on PROMs were not collected pre-operatively. Thus, it was not possible to assess the improvement from baseline to the last follow-up. Furthermore, long-term clinical data concerning the efficacy and safety of AMIC are not yet available. The limitation of missing long-term data generally applies to the majority of treatments, except for primary osteochondral defects treated with arthroscopic BMS produces^44^. The reported outcomes after repeat AMIC and number of secondary procedures related to hardware removal reflect that the treatment of revision AMIC combined with cancellous bone grafting, if necessary, is a viable option, but may benefit from further developments. These innovative modifications may involve the avoidance of a malleolar osteotomy^36^, the less invasive penetration of the subchondral bone^45^, and the augmentation with stem cells (e.g. MAST technique)^39^, among others. In the light of the discussed literature, each available revision technique has an individual profile of safety, efficacy, invasiveness, donor site morbidity, and durability. Ahead of any revision procedure, a comprehensive analysis of the failure is mandatory to match difficult-to-treat patients and lesions with an appropriate surgical revision procedure. The MOCART score was used; however, the reliability of imaging to assess the clinical outcome after cartilage repair has been criticized^46–49^. During the index procedure, 12 patients (44%) received an additional autologous cancellous bone transplantation from the osteotomy site which not healed. The remaining patients received isolated AMIC for chondral defects. Apparently, these patients developed subchondral chondral bone necrosis postoperatively. Whether this may influence the outcome is unclear; however, given the limited sample size included in the present investigation, further considerations or subgroup analyses were not conducted. Most patients underwent conservative management before undertaking revision surgery using AMIC. However, given the heterogeneous nature and the lack of documentation on treatments, further subgroup analyses cannot be conducted. Most patients could not attribute the onset of symptoms to a definite event, declaring to have never fully recovered following the index AMIC. Therefore, the time to failure following the index surgery has not been assessed in the present investigation.

## Conclusion

AMIC could be a viable option as revision procedure for failed AMIC in recurrent symptomatic osteochondral defects of the talus. At approximately four years follow-up, the PROMs indicated that patients were moderately satisfied with the procedure, and the MOCART score demonstrated a significant improvement from baseline to the last follow-up. A deeper understanding in prognostic factors and patient selection is critical to prevent failures.

## Data Availability

The datasets generated during and/or analysed during the current study are available throughout the manuscript.

## Abbreviations

AMIC: Autologous matrix induced chondrogenesis
MD: Mean difference
SE: Standard error
CI: Confidence interval
VAS: Visual Analogic Scale
AOFAS: American Orthopedic Foot and Ankle Score
MOCART: Magnetic resonance observation of cartilage repair tissue
